# Older Victims of Mass Marketing Scams: An Analysis of Data Seized From Scammers

**DOI:** 10.1093/geroni/igab046.1257

**Published:** 2021-12-17

**Authors:** Marguerite DeLiema, Lynn Langton

**Affiliations:** 1 University of Minnesota, Twin Cities, SAINT PAUL, Minnesota, United States; 2 RTI International, RTI International, District of Columbia, United States

## Abstract

Mass marketing scams are some of the most common frauds in America, and include scams perpetrated through the mail. A growing body of research indicates that older adults face a greater risk of victimization due to age-related changes in cognitive functioning and social isolation, and may be more likely to fall victim repeatedly. The aim of this study is to determine the frequency of repeat mass marketing fraud (revictimization) among older adults and patterns of victimization associated with age, scam type, seasonality, and geography. We use two decades of non-public administrative data from the United States Postal Inspection Service (USPIS). These databases were seized during law enforcement investigations of mass mailing scam organizations and contain more than 2 million unique U.S. victims and their transactions with four different fraud organizations. Victims were matched across datasets using name, address, and a change of address file. We find that revictimization rates increase with age in psychic scams. The 10,000 victims who responded the most times (between 82 and 562 times) were 78 years old on average and suffered $4,700 in total losses per person. Other significant trends emerged for lottery and sweepstakes scams. Unlike prior fraud victimization studies, inferences on victim characteristics are based on actual victim experiences with fraud rather than hypothetical scenarios or surveys where victims must self-report fraud. Findings provide valuable policy-relevant information regarding older victims and the patterns of chronic victimization.

